# High tolerance to high-light conditions for the protected species *Ariocarpus kotschoubeyanus* (Cactaceae)

**DOI:** 10.1093/conphys/cox042

**Published:** 2017-07-17

**Authors:** Erika Arroyo-Pérez, Joel Flores, Claudia González-Salvatierra, María L. Matías-Palafox, Cecilia Jiménez-Sierra

**Affiliations:** 1 Departamento de Biología, Universidad Autónoma Metropolitana-Iztapalapa, Av. San Rafael Atlixco 186, Col. Vicentina Iztapalapa, Ciudad de México, C.P. 09340, Mexico; 2 División de Ciencias Ambientales, Instituto Potosino de Investigación Científica y Tecnológica, Camino a la Presa San José No. 2055, Colonia Lomas 4a. Sección, San Luis Potosí, S.L.P., C.P. 78216, Mexico; 3 Facultad de Agronomía y Veterinaria, Universidad Autónoma de San Luis Potosí, Carretera San Luis Potosí Km. 14.5, Soledad de Graciano Sánchez, San Luis, S.L.P., C.P. 78321, Mexico

**Keywords:** Chihuahuan desert, chlorophyll fluorescence, endangered species, living rock cactus, nurse plants, stress tolerance

## Abstract

We determined the seasonal ecophysiological performance under perennial plants and under high solar radiation for adult individuals from the ‘living rock’ cactus *Ariocarpus kotschoubeyanus*, which occurs equally under nurse plants and in open spaces. We evaluated the effective quantum yield of photosystem II (Φ_PSII_) and the dissipation of thermal energy [non-photochemical quenching (NPQ)] thorough the year. The maximum apparent electron transport rate (ETR_max_) and the saturating photosynthetically active photon flux density for PSII (PFD_sat_) were also determined from rapid light curves. We found that although the Φ_PSII_ was higher in shaded sites under potential nurse plants than in exposed sites, all values were close to the optimal value of 0.83. The high Φ_PSII_ found for *A. kotschoubeyanus* plants suggests that they use a great proportion of the absorbed light for photosynthesis, under nurse plants as well as in open spaces. We also found higher NPQ values in exposed sites than in shaded ones but only in Autumn, thus reducing the risk of photoinhibition. In addition, the PFD_sat_ was higher in exposed sites than in shaded ones in Spring, Summer and Autumn, but in Winter there were no differences between treatments. We also found high saturating light levels for ETR (PFD_sat_ higher than 1378 μmol m^−2^ s^−1^) in all seasons but in winter for shaded and non-shaded plants. Our findings indicate that *A. kotschoubeyanus* plants use a great proportion of the light that they absorb for photosynthesis. This high tolerance to high-light conditions could explain why *A. kotschoubeyanus* do not show preferences for protected sites under nurse plants.

## Teaser

In this study, we found a high tolerance to high-light conditions for the protected cactus *Ariocarpus kotschoubeyanus*, which could explain why this species does not show preferences to protected sites under nurse plants.

## Introduction

In response to harsh conditions, many species appear to be more frequent under canopies of adult plants of other species which provide a less stressful micro-environment (Ellner and Shmida, 1981). This association has been called ‘nurse plant syndrome’ (Niering *et al.*, 1963) or ‘nurse–protégé’ interaction (Cody, 1993). Cactaceae is a plant family in which many species grow primarily under nurse plants (Flores and Jurado, 2003). Relationships can vary between cactus species and environments and perhaps multiple causes could be involved in facilitation by nurse plants (Valiente-Banuet *et al.*, 1991; Muro-Pérez *et al.*, 2012). Thus, shade can be beneficial by reducing overheating, excessive transpiration and photoinhibition that plants growing in open areas may experience (Flores and Jurado, 2003; Pérez-Sánchez *et al.*, 2015). However, shade may also represent a cost for the cacti in terms of photosynthetically active radiation because it could induce stress by limiting photosynthesis and arrest plant development (Kitajima and Fenner, 2000). Some cactus species, however, occur equally under nurse plants and in open spaces (Jurado *et al.*, 2013), for which the mechanisms avoiding photoinhibition are unknown.

Photoinhibition is defined as any downregulation of the photosynthetic apparatus in response to excess light when more sugar is produced in leaves than can be utilized by the rest of the plant and/or more light energy is harvested than can be utilized by the chloroplasts for the fixation of carbon dioxide into sugars (Adams *et al.*, 2013). Stress caused by drought or extreme temperatures increases the risk and severity of photoinhibition in arid environments (Valladares, 2004).

*Ariocarpus kotschoubeyanus* (Lem.) K. Schum (Cactaceae) is an especially protected species in the framework of the environmental laws and regulations of México (Semarnat, 2010), and as near threatened in the framework of the international regulations of IUCN (Gómez-Hinostrosa *et al.*, 2013), as well and is listed under Appendix 1 of CITES (Sajeva *et al.*, 2012). This species occurs equally under nurse plants and in open spaces at the southern part of its distribution (Suzán-Aspiri *et al.*, 2011). It is unknown if this lack of micro-site preference occurs across the species’ entire climate range. Thus, we hypothesized that at the southern part of its distribution *A. kotschoubeyanus* performs better under nurse plants, showing higher maximum quantum yield of photosystem II (Φ_PSII_) and electron transport rate (ETR) values, under nurse plants than individuals under direct sunlight, but higher non-photochemical quenching (NPQ) in open spaces than under nurse plants in order to tolerate stress. We also hypothesized that the light level at which *A. kotschoubeyanus* PSII becomes saturated (PPFD_sat_) is related to acclimatization to the light environment in which the plants grew. Thus, if plants grow in open sites then they must show high saturating light levels for ETR during all year.

## Materials and methods

### Study site

The study site includes one population of *A. kotschoubeyanus* located in Tolimán, Querétaro, México (latitude 20°52′N; longitude −99°57′ W; 1 200 msnm) at the southern part of the Chihuahua desert. This area has an annual temperature of 19.2°C and an annual precipitation of 361.4 mm (CONAGUA, 2016). Its vegetation type is thorny xerophilous scrub (González-Medrano, 2012).

### Studied species

*Ariocarpus kotschoubeyanus* (Lem.) K. Schum (Cactaceae) is a globose-depressed cactus that grows in the Chihuahuan Desert from western Coahuila through Querétaro; it can reach 7 cm in diameter (Pilbeam and Weightman, 2006). This species is called ‘living rock cactus’, like all *Ariocarpus* species (Glass and Foster, 1974). As other *Ariocarpus*, this species presents triangular flattened tubercles and is usually found semi-buried during periods of drought (Bravo-Hollis and Sánchez-Mejorada, 1991; Anderson, 2001). Populations of *A. kotschoubeyanus* are threatened due to increased agriculture and livestock areas, urban expansion, residue deposits and overexploitation for medicinal or ornamental purposes (Anderson *et al.*, 1994; Oldfield, 1997).

### Micro-environmental measurements

Photon flux density (PFD) and temperature were registered at midday using a portable pulse amplitude modulation fluorometer (Mini-PAM; H. Walz, Effeltrich, Germany). PFD was measured with a micro-quantum sensor (0.5 mm diameter), and temperature was evaluated with the aid of a NiCr-Ni thermocouple, both measurements were done at the photosynthetic surface of the stem (de la Rosa-Manzano *et al.*, 2016). Measurements were conducted under nurse plants and in open sunlight during periods of full sunlight. All measurements were performed once in each season (Autumn 2011, and Winter, Spring, and Summer 2012).

### Chlorophyll fluorescence of *A. kotschoubeyanus* in open species and under nurse plants

We selected *A. kotschoubeyanus* adult plants of 3–6 cm diameter and without damaged tubercles. *Karwinskia humboldtiana* (Schult.) Zucc. (Rhamnaceae), a common species in the study area, was chosen as nurse plant. Thus, *A. kotschoubeyanus* plants under *K. humboldtiana* individuals (one cactus per nurse plant, *n* = 6) and six *A. kotschoubeyanus* plants in exposed sites (*n* = 6) were selected. Shaded plants were under the densest part of the shrub canopy, near the stem of the nurse plant. The exposed plants were not shaded by nearby shrubs or rocks.

The effective quantum efficiency of photosystem II (Φ_PSII_), the ETR, and the NPQ in exposed plants (*n* = 6) and under the more frequent nurse plant found (*n* = 6), were measured after acclimation in darkness for 20 min once each year season. All measures were performed once by each season using a portable fluorometer (Mini-PAM, Walz, Effeltrich, Germany). The Mini-PAM was equipped with a leaf-clip holder (2030-B; Walz), where the optic fibre was inserted; the distance between the optic fiber and the surface stem was ~12 mm, with an angle of 60° relative to the upper surface of the stem.

The light source was a halogen lamp inside the instrument. The intensity of actinic light was increased every 10 s for 2 min. Photosynthetic PFD and temperature data were used to estimate a series of variables related to the photosynthetic performance of plants located under nurse plants and outside them. Chlorophyll fluorescence measurements was carried out at noon (between 12:00 and 14:00 h), when plants faced the maximum daily temperature. The effective quantum efficiency of photosystem II (Φ_PSII_) was estimated as (*F*′_m_– *F*_t_)/*F*′_m_, where *F*_t_ is the chlorophyll fluorescence emitted by plants under steady-state illumination (i.e. light conditions in the field) and *F*′_m_ is the maximum fluorescence emitted by chlorophyll when a saturating pulse of actinic light is superimposed to environmental levels of light (Genty *et al.*, 1989). The values for Φ_PSII_ oscillate between 0.80 and 0.83 if environmental stress is negligible for plants, but these values decreased with increasing environmental stress (Maxwell and Johnson, 2000).

The ETR across the electron chain of chloroplasts was estimated as ETR = Φ_PSII_ × PFD × 0.84 × 0.5, where Φ_PSII_ is the effective quantum yield of photosystem II, PFD is the photosynthetic PFD recorded by the sensor in the leaf clip, 0.84 is the estimated mean proportion of incident light absorbed by the photosystems (Ehleringer, 1981) and 0.5 is the required factor for both photosystems to account for absorbed photons (Roberts *et al.*, 1996).

Finally, we calculated the NPQ efficiency. This variable was calculated as $\mathrm{NPQ}=(F_{o}-F_{m}^{\prime })/F_{m}^{\prime }$, where $F_{o}$ is the basal chlorophyll fluorescence emitted by cacti at darkness, and $F_{m}^{\prime }$ is the maximum fluorescence emitted by chlorophyll after imposing a saturating pulse of actinic light at noon. NPQ specifically refers to the mechanism used by plants to dissipate the excess of light energy captured by chlorophylls as heat. This mechanism of energy dissipation is linked to the xanthophyll cycle, and high NPQ values are expected with increasing levels of environmental stress (Maxwell and Johnson, 2000; Aragón-Gastélum *et al.*, 2014).

In order to evaluate the level of light at which photosystem II is saturated (PFD_sat_) (Rascher *et al.*, 2000; Hernández and Briones-Villarreal, 2007), rapid light curves (RLC) for chlorophyll fluorescence were produced. Light curves allow to deduce cardinal points which are quantitative physiological indicators of intrinsic photosynthetic capacity (Lüttge and Scarano, 2007), such as the maximum apparent ETR (ETR_max_) and the saturating photosynthetically active PFD for PSII (PFD_sat_).

For each species, the data for Φ_PSII_ and ETR against PFD were adjusted according to the statistical models proposed by Rascher *et al.* (2000). With the adjusted ETR vs. PFD curve, the cardinal points were determined: ETR_max_ and saturating photosynthetically active PFD for PSII (PFD_sat_), determined to 0.9 of ETR_max_. The RLC were produced using two scales of the Mini-PAM light curve program to obtain a sequence of 0, 255, 399, 590, 807, 1184, 1587 and 2372 μmol of PFD m^−2^ s^−1^. These RLC were obtained in exposed plants (*n* = 6) and under the more frequent nurse plant found (*n* = 6), once each year season.

### Statistical analysis

Factorial ANOVAs for repeated measurements were carried out for Φ_PSII_, NPQ and ETR_max_, as well as for temperature and photosynthetic PFD, using micro-environment as factor. There were two micro-environment levels (under nurse plant an under direct sunlight) and four season levels (Spring, Summer, Autumn and Winter). Tukey tests were used to detect different means. Analyses were carried out using STATISTICA (8) with *α* = 0.05. Data were transformed, if required to comply with the assumption of normal distribution (Sokal and Rohlf, 1995).

## Results

### Micro-environmental measurements

**Figure 1:**
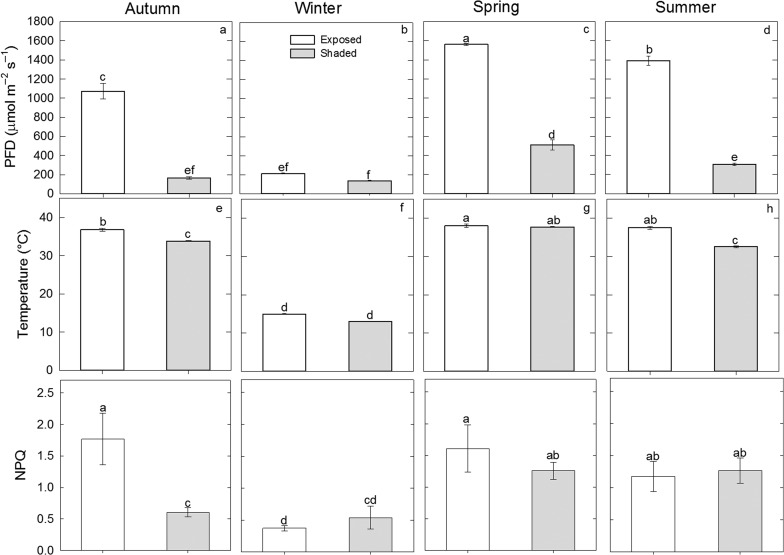
Photon flux density (PFD, μmol m^−2^ s^−1^), temperature (°C) and maximum quantum yield of photosystem II (*F*_v_/*F*_m_) of *Ariocarpus kotschoubeyanus* under nurse plants and in exposed areas during the 4-year seasons. Data are means ± SE, *n* = 6. Different letters represent significant differences between the interaction micro-environment × season (*P* < 0.05).

The photosynthetic PFD was affected by the micro-environment (*F*_1,10_ = 667.69; *P* < 0.00001), the season (*F*_3,30_ = 188.08; *P* < 0.00001), and the interaction micro-environment X season (*F*_3,30_ = 72.16; *P* < 0.00001). The PFD was lower under shaded sites nurse plants than in exposed sites at all seasons except for Winter (Fig. 1a–d). Higher typical daily PFD in open spaces was found in Spring than in Summer, as well as in Summer than in Autumn. The lowest PFD was found in Winter (Fig. 1a–d).

Temperature was affected by the micro-environment (*F*_1,10_ = 93.37; *P* < 0.00001), the season (*F*_3,30_ = 4276.58; *P* < 0.00001) and the interaction micro-environment × season was significant (*F*_3,30_ = 31.71; *P* < 0.00001). The temperature was lower under nurse plants than in exposed sites at Summer and Autumn. The lowest temperature was found in Winter in both nurse plants and open spaces (Fig. 1e–h).

### Chlorophyll fluorescence of *A. kotschoubeyanus* in open species and under potential nurse plants

The effective quantum yield of photosystem II (Φ_PSII_) was affected by micro-environment (*F*_1,10_ = 9.89; *P* = 0.01), but not by the season (*F*_3,30_ = 1.40; *P* = 0.26), or by the micro-environment × season interaction (*F*_3,30_ = 1.68; *P* = 0.192). In average for all seasons, the Φ_PSII_ was higher in shaded sites under potential nurse plants (0.80 ± 0.01) than in exposed sites (0.75 ± 0.03).

NPQ values were affected by the season (*F*_3,30_ = 6.34; *P* = 0.001), and by the micro-environment × season interaction (*F*_3,30_ = 3.22; *P* = 0.032), but not by the micro-environment (*F*_1,10_ = 3.46; *P* = 0.07). We found higher NPQ values in exposed sites than in shaded ones only in Autumn (1.76 ± 0.41 SE vs. 0.60 ± 0.07, respectively; Fig. 1i). No significant differences in the NPQ values between treatments were found in the other seasons, but in general it was lower in Winter (Fig. 1i–l).

**Figure 2:**
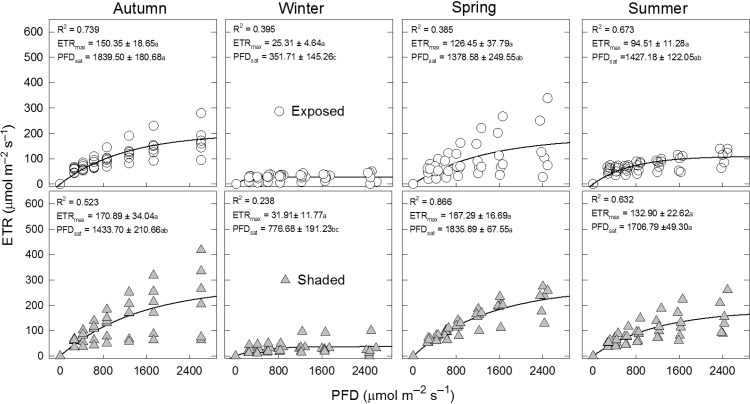
Rapid light curves determined from the maximum apparent electron transport rate (ETR_max_) and the saturating photosynthetically active photon flux density for PSII (PFD), by adjusting an exponential function using the Sigma Plot Program. Data are means ± SE, *n* = 6. For PFDsat, different letters represent significant differences between the interaction micro-environment × season (*P* < 0.05). For ETR_max_, this interaction was not significantly affected (*P* > 0.05).

The ETR_max_ was affected by the season (*F*_3,30_ = 13.67; *P* < 0.00001) and by the micro-environment (*F*_1,10_ = 5.65; *P* = 0.038), but not by the interaction micro-environment × season (*F*_3,30_ = 0.49; *P* = 0.68; Fig. 2). *A. kotschoubeyanus* had lower values in Winter (28.61 ± 6.11) than in Spring (156.87 ± 21.72), Summer (113.70 ± 13.07) and Autumn (160.62 ± 18.76); as well as lower ETR_max_ values in exposed sites (99.15 ± 14.16) than under nurse plants (130.75 ± 16.49).

The saturated photosynthetic PFD (PFD_sat_) was affected by season (*F*_3,30_ = 20.55; *P* < 0.00001), and by the micro-environment × season interaction (*F*_3,30_ = 3.09; *P* = 0.04; Fig. 2), but not by the micro-environment (*F*_1,10_ = 3.07; *P* = 0.11). In general, PFD_sat_ was higher in Spring, Summer and Autumn than in Winter. In addition, PFD_sat_ was higher in exposed sites than in shaded ones in Spring, Summer and Autumn, but in Winter there were no differences between treatments (Fig. 2).

## Discussion

Cacti have been subjected to intensive exploitation due to their great value, mainly as ornamental plants, thus their populations have been drastically affected due to illegal collection and habitat destruction (Anderson *et al.*, 1994; Sajeva *et al.*, 2012). As suggested by Brussard (1991), the collection of basic life-history information, including the influence of environmental factors on development, can be very useful in the conservation of rare species such as *A. kotschoubeyanus*. Similarly, Wikelski and Cook (2006) suggested that, for conservation strategies to be successful, it is important to understand the physiological responses of organisms to their changing environments. More recently, Cooke *et al.* (2013) mentioned that physiological tools and knowledge are especially useful for developing cause and effect relationships, and for identifying the optimal range of habitats and stressor thresholds for different organisms. Thus, by knowing the ecophysiological responses in the different micro-environments where these species occur, we can better understand the micro-environment to properly manage this and other endangered cacti.

Measurements of light–response curves lead to a deeper insight into characteristic parameters of an investigated plant, which are not related to the momentary ambient light conditions, but rather to the ontogeny of a photosynthetic shoot and to the range of physiological plasticity of a plant. Therefore the so-called cardinal points of light–response curves are highly interesting in ecophysiological research (Rascher *et al.*, 2000).

We hypothesized that although *A. kotschoubeyanus* shows lower Φ_PSII_ and ETR values under direct sunlight than under nurse plants, it shows higher NPQ values under direct sunlight in order to tolerate stress. Our hypothesis was supported in that we found higher Φ_PSII_ and ETR_max_ in shaded sites under potential nurse plants than in exposed sites, although all values were close to the optimal value of 0.83 (Maxwell and Johnson, 2000). Our Φ_PSII_ values (0.80 ± 0.01 for plants under nurse plants and 0.75 ± 0.03 for plants in exposed sites) appear to be higher than those found for other cacti (Hernández-González and Briones Villarreal, 2007; Badano *et al.*, 2016). These high Φ_PSII_ values suggest that *A. kotschoubeyanus* plants use a great proportion of the light that they absorb for photosynthesis, such under nurse plants as in open spaces.

Higher Φ_PSII_ values in shaded sites compared to exposed sites have also been found for cactus seedlings by Hernández-González and Briones-Villarreal (2007). These authors found that the Φ_PSII_ of 1-week old *Pachycereus weberi* and *Escontria chiotilla* seedlings was higher in the shade than in high-light. Pérez-Sánchez *et al.* (2015) also found that Φ_PSII_ values of seedlings from seven succulent species, four cacti (*Echinocactus platyacanthus*, *Ferocactus histrix*, *Myrtillocactus geometrizans* and *Stenocactus coptonogonus*) and three Asparagaceae (*Agave lechuguilla*, *Agave salmiana* and *Yucca filifera*) were greater under nurse plants than in open spaces.

Hernández-González and Briones-Villarreal (2007) also found similar values of Φ_PSII_ in the seedlings and adults of *Stenocereus stellatus*, *M. geometrizans* and *Ferocactus recurvus* (Φ_PSII_ = 0.61 on average), even though the PFD was >2000 μmol m^−2^s^−1^ in the field and 1500 or 750 μmol m^−2^ s^−1^ under high-light or shade. In contrast, adult plants of *P. weberi* and *E. chiotilla* had high Φ_PSII_ (0.68 on average) in the field, while their seedlings had lower (Φ_PSII_ = 0.52 on average) in the shade.

The Φ_PSII_ has also been analysed for adult individuals of *Cylindropuntia leptocaulis* (Cactaceae) located under the canopy of *Larrea tridentata* and in open sites (Badano *et al.*, 2016). In this study, at 15:00 h higher Φ_PSII_ in shaded sites under nurse plants than in exposed sites was found.

We expected higher NPQ in open spaces than under nurse plants to tolerate stress and thus reduce the risk of photoinhibition (Adams *et al.*, 1987; Barker and Adams, 1997; Barker *et al.*, 1998; Aragón-Gastélum *et al.*, 2014), but NPQ values were similar in exposed sites and under nurse plants in all seasons but Autumn, in which higher NPQ values were found in exposed sites than in shaded ones. Similarly, the NPQ values for cactus seedlings in high-light and shade were generally low, and differences were not statistically significant (Hernández-González and Briones-Villarreal, 2007). The Φ_PSII_ values close to the optimal and similar NPQ values between plants in open sites and under nurse plants are findings helping to explain why *A. kotschoubeyanus* does not show association with nurse plants (Suzán-Azpiri *et al.*, 2011).

Because high ETR values indicate increased photosynthetic performance in plants (Ritchie and Bunthawin, 2010; Aragón-Gastélum *et al.*, 2014; Pérez-Sánchez *et al.*, 2015), we also hypothesized that *A. kotschoubeyanus* shows high saturating light levels for ETR during all year. This hypothesis was supported in that we found high saturating light levels for ETR (PFD_sat_ higher than 1378 μmol m^−2^ s^−1^ in both under nurse plants and in exposed sites) in most seasons, but in Winter in where ETR becomes saturated at lower flux densities of light (PFD_sat_ = 351.71 μmol m^−2^ s^−1^ in exposed sites and 776.68 μmol m^−2^ s^−1^ under nurse plants). Low ETR values in Winter coincided with low NPQ values. These high saturating light levels for ETR suggest high tolerance to high-light conditions could also explain that *A. kotschoubeyanus* does not show preferences by protected sites under nurse plants (Suzán-Azpiri *et al.*, 2011).

## Conclusions

Our work provided strong evidence about how the cactus *A. kotschoubeyanus* tolerates the high-light intensities occurring in its habitat. This is the first study evaluating physiological performance for adult cacti under perennial species and under high solar radiation. Our findings give us a better understanding of the mechanisms that cacti use to survive under environmental stresses, which can be useful for conservation and management practices of this species and other endangered cacti.
